# High regional mortality due to malignant melanoma in Eastern Finland may be explained by the increase in aggressive melanoma types

**DOI:** 10.1186/s12885-021-08879-1

**Published:** 2021-10-29

**Authors:** Ville Suhonen, Jaana Rummukainen, Hanna Siiskonen, Arto Mannermaa, Ilkka T. Harvima

**Affiliations:** 1 Department of Dermatology, University of Eastern Finland and Kuopio University Hospital, P.O.B. 100, 70210 Kuopio, Finland; 2 Department of Pathology, Kuopio University Hospital, 70210 Kuopio, Finland; 3 Department of Pathology, University of Eastern Finland, 70210 Kuopio, Finland; 4 Biobank of Eastern Finland, Kuopio University Hospital, 70210 Kuopio, Finland

**Keywords:** Melanoma, Risk factor, Metastasis, Mortality, Prognosis, Immunosuppression

## Abstract

**Background:**

A regional skin cancer prevention program in Eastern Finland revealed a relatively high age-standardized mortality due to malignant melanoma during 2013–2017. An explanation for this is needed.

**Purpose:**

To analyse the 543 melanoma samples in 524 subjects collected during 2000–2013 at Kuopio University Hospital and reposited in the Biobank of Eastern Finland. A focus was directed to factors related to metastasis.

**Methods:**

The samples were analysed anonymously by examining the histopathological report, referral text and the list of diagnoses. A possible state of immunosuppression was evaluated.

**Results:**

The mean age at the diagnosis of malignant melanoma (MM), lentigo maligna (LM) and melanoma in situ was relatively high, i.e., 66.2, 72.1 and 63.3, respectively. Especially the MM type increased markedly during 2000–2013. In further analyses of a representative cohort of 337 samples, the proportion of nodular melanoma and LM/LMM melanoma was relatively high, 35.6 and 22.0%, respectively, but that from superficial spreading melanoma relatively low (33.8%). Metastasis correlated with immunosuppression, male gender, Clark level, Breslow thickness, ulceration, mitosis count, invasion into vessels and/or perineural area, microsatellites, melanoma subtype, body site, recidivism, and the absence of dysplastic nevus cells.

**Conclusion:**

The marked increase in aggressive melanomas with associated metastasis, and the relatively high age at diagnosis, can partially explain the mortality.

**Supplementary Information:**

The online version contains supplementary material available at 10.1186/s12885-021-08879-1.

## Introduction

Cutaneous malignant melanoma is the 5th most common tissue cancer in Finland (GLOBOCAN 2020, http://gco.iarc.fi/today) and its incidence, together with the most common type of tissue cancer, non-melanoma skin cancer (NMSC, keratinocyte skin cancer), has been increasing steadily in Western countries [1, 2]. Worldwide, the incidence of melanoma is higher in the fair-skinned population [2–4]. The incidence of melanoma in the Nordic countries of Europe is higher than that in the Baltic states [3, 4]. In Finland, the male/female incidence ratio of melanoma has been reported to be 1.1 differing from the corresponding ratio in Sweden (1.0), Norway (0.9), Denmark (0.8), Estonia (0.8), Latvia (0.7), Lithuania (0.7) and Iceland (0.6) [3].

Melanoma presents as various clinical phenotypes, including melanoma in situ and its variant lentigo maligna (LM), lentigo maligna melanoma (LMM), superficial spreading melanoma (SSM), nodular melanoma (NM), acral lentiginous melanoma (ALM), amelanotic melanoma (AM) and desmoplastic melanoma [5]. The traditional prognostic factors still constitute the basis of the management and are associated with metastasis, and the Breslow thickness and ulceration are still the determining tumor-related factors in the 8th AJCC edition of TNM staging of melanoma, while the mitotic rate is no longer needed to determine the T category [2, 5, 6]. However, other prognostic factors have also been identified [2, 7–9].

Within the country of Finland, there is surprisingly marked regional variation in the age-standardized incidence and mortality rates due to melanoma during 2013–2017 (Table 1). The mortality rate is comparably high in Central and Eastern Finland, but especially so in the hospital district of Northern Savo located in Eastern Finland with a population of about 251,000 (Table 1). The reasons for these regional differences may be related to behavioral or population differences, general morbidity (Table 1), treatment modalities, activity in cancer reporting or screening, and/or differences in tumor features. To find an explanation for the high mortality in this hospital district with Kuopio University Hospital as the referral hospital, this study had the unique possibility to utilize all the tissue samples collected at Kuopio University Hospital during 2000–2013. The melanoma samples were identified, typed and examined for factors related to metastasis.

**Table 1:** The age-standardized incidence and mortality rates of cutaneous malignant melanoma for male and female subjects during 2013–2017 as well as the age-standardized morbidity index during 2014–2016 in 5 university hospital regions in Finland

Region in the country	Incidence* Males	Incidence* Females	Mortality* Males	Mortality* Females	Morbidity index**
Southern Finland (Helsinki University Central Hospital)	41/100,000	32/100,000	6.8/100,000	2.7/100,000	86.1
South-Western Finland (Turku and Tampere University Hospitals)	30/100,000	25/100,000	6.6/100,000	2.1/100,000	94.2
	37/100,000	29/100,000	6.6/100,000	2.1/100,000	101.8
Northern Finland (Oulu University Hospital)	22/100,000	15/100,000	5.3/100,000	1.8/100,000	117.7
Central and Eastern Finland (Kuopio University Hospital)	24/100,000	18/100,000	5.7/100,000	2.7/100,000	118.5
- hospital district of Northern Savo	25/100,000	19/100,000	6.6/100,000	3.1/100,000	130.0

“*”: Public data retrieved from the Finnish Cancer Registry; “**”: public data retrieved from the Finnish Institute for Health and Welfare. The morbidity index is measured by taking into account of 7 different disease groups: cancer, coronary artery disease, cerebrovascular diseases, musculoskeletal diseases, mental health disorders, accidental injuries and dementia (https://thl.fi/en/web/thlfi-en/statistics/statistics-by-topic/morbidity/thl-s-morbidity-index)

## Materials and methods

### Subjects and methods

All the tissue samples (285,360 from 70,420 individual patients) collected at Kuopio University Hospital during the treatment and diagnostic procedures of patients during January 1st, 2000, through August 31st, 2013, have been transferred to the Biobank of Eastern Finland to be used for health research according to the national Biobank Law. Based on this material, a total of 524 patients with 543 melanomas were identified and classified according to the codes of Systematized Nomenclature of Medicine (SNOMED, http://snomed.org): M87422 = lentigo maligna (LM, *n* = 109); M87202 = melanoma in situ (Clark I) (MIS, *n* = 52), and M87203 = malignant melanoma (MM, *n* = 382).

In further analyses, 337 primarily diagnosed melanomas (cohort 2), selected from the 524 subjects (cohort 1), were included in the analysis based on the sufficient amount of information available from the referral text and pathology report. The information was collected from the QPati software (Tieto® QPati®, Effica Patologia, Version 3.0.3.10) of the Department of Pathology of Kuopio University Hospital by reading the referral and histopathological reports. The most representative melanoma sample, either a biopsy or an excised tumor, was selected from each patient. The official list of all diagnoses from the medical record of each patient provided by the biobank was examined and followed-up until the date of data retrieval, May 24th, 2019. Therefore, there was at least a 5-year follow-up time after melanoma diagnosis to clarify possible metastasis. The distribution of the 3 SNOMED melanoma types was almost equal in these 337 cases, LM (*n* = 75), MIS (*n* = 35), and MM (*n* = 227), compared to that in 543 samples. Thus, this cohort represents reasonably well the total of 543 melanoma samples.

The cohort of 337 melanoma samples was divided to following subtypes: SSM, NM, LM/LMM, ALM. The clinicopathological parameters were as follows: the age at the time of diagnosis, body site, gender, immunosuppressive medication, the list of diagnoses, organ transplantation, prevalence of dysplastic nevi, Breslow thickness, Clark level, ulceration, growth type, metastasis, recidivism (data available from 337 cases), regression (335), number of mitoses (224), presence of benign nevus cell islands (apart from the tumor) and the presence or absence of dysplastic nevus cells (DN^+^/ DN^−^) (146), invasion into the lymphatic/blood vessels or perineural area (103), cell type (101) and microsatellites (30). A diagnosis of immune-mediated disease in any organ (*n* = 34, including autoimmune connective tissue or skin blistering diseases, chronic inflammatory diseases of the gastrointestinal tract or central nervous system, rheumatic joint diseases, systemic vasculitis, sarcoidosis, lymphoma, leukemia and myeloma), organ transplantation (*n* = 5, during 1988–2009) or information on any marked systemic immunosuppressive medication (*n* = 136, including systemic corticosteroids, cytostatic drugs, ciclosporin, tacrolimus, mycophenolate mofetil, TNF-alpha blockers) was suggestive for an immunosuppressive state and the subjects were classified into immunosuppressed and non-immunosuppressed groups accordingly. Mitosis count was defined verbally (*none, little, a lot* = at least 1 mitosis per square millimeter), because in some of the reports mitosis count was mentioned only verbally. For Breslow thickness, a following classification was used: ≤1.00 mm (class 1), 1.01–2.00 mm (2), 2.01–4.00 (3) and > 4.00 mm (4). Breslow thickness could be clarified from 467 out of 543 samples and the distributions were very similar with those in the cohort of 337 samples and no significant difference was seen (*p* = 0.637).

National Supervisory Authority for Welfare and Health (Valvira) and the Ethics Committee of research at Kuopio University Hospital have given the authorization to transfer the samples and related information from Kuopio University Hospital to the Biobank of Eastern Finland. The research plan was approved by the scientific committee of the Biobank of Eastern Finland. The samples and patients in this study were analysed anonymously without an access to detailed medical records.

### Statistics

The material was analysed using the Chi-square test and the binary logistic regression analysis with SPSS software (IBM® SPSS® Statistics, Version 27, 64-bit edition). In the binary logistic regression analysis, the parameters with over 300 observations and significance in the Chi-square test were tested to identify the independent factors related to metastasis. For the age in the binary logistic regression analysis, the cohort was divided into two age groups according to the ROC curve analysis producing the highest specificity and sensitivity for metastasis at the cut-off age of 66.8 years.

## Results

### The incidence of melanoma and its subtypes during 2000–2013

**Fig. 1:**
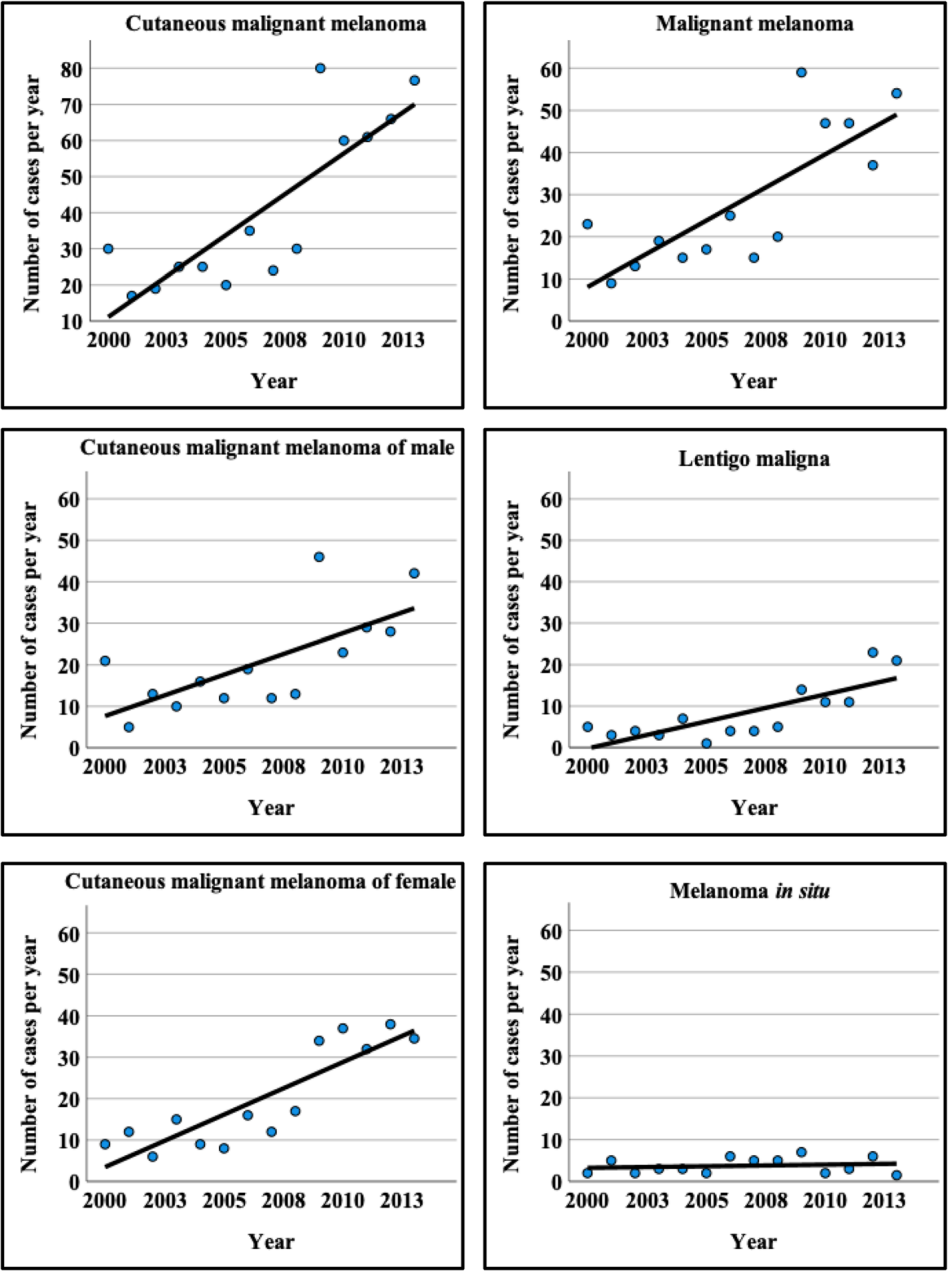
The number of melanoma cases during 2000–2013. The year 2013 was extrapolated to the end of December 31st

The absolute increase of melanomas in 524 patients (50.4% males and 49.6% females) with 543 melanomas was 4.5 cases per year (*p* < 0.001), and the mean age at diagnosis did not change significantly during 2000–2013. For males (*n* = 275), it was 2.0 cases (*p* = 0.006), and for females (*n* = 268) 2.5 cases per year (*p* < 0.001) (Fig. 1). To clarify whether the malignant or in situ subtype of melanoma increased, the samples were classified according to the SNOMED diagnosis codes to M87203 = MM, M87422 = LM, and M87202 = MIS. The age of subjects at diagnosis was 66.2 ± 14.6 for MM, 72.1 ± 12.2 for LM and 63.3 ± 16.4 for MIS. The age of 337 subjects with 345 melanomas at diagnosis was 66.1 ± 15.6 for MM, 74.2 ± 11.6 for LM and 66.6 ± 14.1 for MIS, i.e., similar to that in 524 cases with 543 samples.

MM and LM showed an increase of 3.1 (*p* < 0.001) and 1.3 cases per year (*p* < 0.001), respectively. For MIS, there was no significant rise (Fig. 1). MM increased more in females (2.0 cases per year, *p* < 0.001) than in males (1.2 cases per year, *p* = 0.022). In contrast, the increase in LM was 0.8 cases per year (*p* = 0.001) in males and 0.5 cases per year in females (*p* = 0.011). In both genders, the increase was non-significant in MIS. The population in this hospital district of Northern Savo decreased from 259,639 in 2000 to 253,643 in 2013 (Statistics Finland). In addition, based on the public data in Finnish Cancer Registry the age-standardized incidence of MM in this hospital district increased from 15.1/100.000 in 2000–2004 to 20.7/100.000 in 2010–2014.

### Melanoma subtypes and anatomical distribution in 337 melanoma patients

A more detailed analysis on tumor characteristics with the most representative melanoma sample was performed in 337 subjects, of whom 53.1% were males and 46.9% females, and 42.1% of the subjects were classified to the immunosuppressed group. The mean age ± SD at diagnosis was 66.6 ± 14.1 years in males (median 68.9 years) and 69.0 ± 15.8 years in females (median 71.3) (*t*-test for the means, *p* = 0.15) (mean in all cases 67.7 ± 14.9, median 69.8, range 10.3–96.8). There was no significant difference in the age between immunosuppressed and non-immunosuppressed subjects.

The largest subtype was NM (35.6%) followed by SSM (33.8%), LM/LMM (22.0%) and ALM (8.6%). The mean age at diagnosis was 74.0 ± 11.5 (LM/LMM), 71.4 ± 13.6 (ALM), 65.9 ± 16.1 (NM) or 64.7 ± 14.7 (SSM). In the samples, 6.2% showed pure vertical growth, 35.6% vertical growth with concomitant radial growth, 24.3% radial growth with concomitant vertical growth, and 33.8% pure radial growth. The proportion of subtypes did not differ between immunosuppressed and non-immunosuppressed groups.

The most common body site was head & neck (HN) followed by trunk, upper limb (UL), lower limb (LL), foot/sole, palm and genital/perianal area. The prevalence of TANS (thorax, upper arm, neck and scalp) melanomas was significantly higher in males than females (with and without LM/LMMs) (Supplementary Table 1) as well as melanomas of the trunk (*p* < 0.001). Females revealed more HN (*p* = 0.007) and LL melanomas (*p* < 0.001) than males. A similar difference between genders was noted for trunk (*p* = 0.004), HN (*p* < 0.001) and LL (*p* = 0.003) melanomas in the non-immunosuppressed group, and for trunk (*p* = 0.001) and LL (*p* = 0.023), but not HN (*p* = 0.514), melanomas in the immunosuppressed group (Table 2). A higher prevalence of UL lesions was noted in the non-immunosuppressed than immunosuppressed group (15.4% vs. 7.7%, *p* = 0.034).

**Table 2:** The difference between female and male subjects with (IS) or without (non-IS) immunosuppression (non-IS) in respect to the body site of melanoma

	Female (158)	Male (179)	p-value	Non-IS Female (97)	Non-IS Male (98)	p-value	IS Female (61)	IS Male (81)	p-value	Total (337)
**HN**	43.0%	29.1%	0.007	47.4%	27.6%	0.004	36.1%	30.9%	0.514	35.6%
**Trunk**	19.0%	46.4%	< 0.001	16.5%	42.9%	< 0.001	23.0%	50.6%	0.001	33.5%
**UL**	12.0%	12.3%	0.941	14.4%	16.3%	0.714	8.2%	7.4%	0.862	12.2%
**LL**	14.6%	3.4%	< 0.001	13.4%	2.0%	0.003	16.4%	4.9%	0.023	8.6%
**Foot/sole**	7.0%	5.6%	0.602	6.2%	6.1%	0.985	8.2%	4.9%	0.430	6.2%
**Palm**	3.2%	1.7%	0.370	2.1%	2.0%	0.992	4.9%	1.2%	0.189	2.4%
**GPA**	1.3%	0.6%	0.490	0.0%	1.0%	0.319	3.3%	0.0%	0.101	0.9%
**N.S.**	0.0%	1.1%	N.D.	0.0%	2.0%	N.D.	0.0%	0.0%	N.D.	0.6%

HN = head & neck; UL = upper limb; LL = lower limb; GPA = genitalia/perianal area; N.S. = not specified; N.D. = no data

### Factors associated with metastasis and other melanoma parameters

Melanoma metastasized in 21.7% of the 337 subjects: into lymph nodes (15.7%) and/or distal organs (12.8%). Melanoma re-appeared in 5.9% of the subjects. Melanoma metastasis correlated with male gender (*p* = 0.001), younger age (*p* = 0.005 in the whole cohort, and *p* = 0.017 without LM/LMMs), immunosuppressive state (*p* = 0.001), Clark level (*p* < 0.001), Breslow thickness (*p* < 0.001), ulceration (*p* < 0.001), mitosis count (*p* < 0.001), invasion into lymphatic/blood vessels or perineural area (*p* < 0.001), the presence of microsatellites (*p* = 0.044), melanoma subtype (*p* < 0.001, in descending order: NM, ALM, SSM and LM/LMM), body site (*p* = 0.022, in descending order: foot & sole, genital/perianal area, trunk, palm, LL, UL and HN) (Table 3), recidivism (50% vs. 19.9%, *p* = 0.002), TANS region (32.8% vs. 14.9%, *p* < 0.001), and DN^−^ (*p* = 0.001) (Supplementary Table 2).

**Table 3:** The association of melanoma parameters with metastasis

	***Metastasis into lymph nodes or distal organs***		***p-value within subgroup***
	***n***	***% within subgroup***	
**CMM subtype**			
**NM**	52/120	43.3%	< 0.001
**ALM**	10/29	34.5%	
**SSM**	11/114	9.6%	
**LM/LMM**	0/74	0.0%	
**Clark**			
**5**	21/28	75.0%	< 0.001
**4**	28/70	40.0%	
**3**	17/83	20.5%	
**2**	5/44	11.4%	
**1**	2/112	1.8%	
**Breslow**			
**4**	27/42	64.3%	< 0.001
**3**	22/47	46.8%	
**2**	12/46	26.1%	
**1**	12/202	5.9%	
**Ulceration**			
**Yes**	27/51	52.9%	< 0.001
**No**	46/286	16.1%	
**Mitosis Count**			
**A lot**	35/83	42.2%	< 0.001
**Little**	20/113	17.7%	
**No Mitosis**	2/28	7.1%	
**LV/BV/PN**			
**Yes**	2/3	66.7%	< 0.001
**No**	7/100	7.0%	
**Microsatellites**			
**Yes**	7/12	58.3%	0.044
**No**	4/18	22.2%	
**Body site**			
**Foot & sole**	8/21	38.1%	0.022
**Genitalia/perianal area**	1/3	33.3%	
**Trunk**	33/113	29.2%	
**Palm**	2/8	25.0%	
**Lower limb**	7/29	24.1%	
**Upper limb**	6/41	14.6%	
**Head & neck**	15/120	12.5%	
**Total**	73/337	21.7%	

NM = nodular; ALM = acral lentiginous; SSM = superficial spreading; and LM/LMM = lentigo maligna/lentigo maligna melanoma; LV/BV/PN = invasion into lymphatic/blood vessels or perineural area

In the binary logistic regression, recidivism (3.9 OR, *p* = 0.039), subtypes (3.6 OR in ALM and NM compared to LM/LMM and SSM, *p* = 0.011), TANS (3.2 OR, *p* = 0.003), Clark level (2.8 OR in melanomas with Clark level 4–5 compared to 1–3, *p* = 0.029), male gender (2.7 OR, *p* = 0.008), age (2.7 OR in subjects below 66.8 years, *p* = 0.006) and Breslow thickness (2.6 OR in melanomas with Breslow class 3–4 compared to 1–2, *p* = 0.043) were independent factors increasing the risk for metastasis (immunosuppression and ulceration remained non-significant, *p*-values 0.054 and 0.171, respectively).

Trunk melanomas showed higher Clark level (*p* < 0.001) and Breslow thickness (*p* = 0.004), contained more NMs (*p* = 0.002) and less LM/LMMs (*p* = 0.001), and displayed more vertical growth types (*p* < 0.001) compared to HN melanomas (percentages not shown). In terms of Breslow thickness (*p* < 0.001), Clark level (*p* < 0.001), ulceration (*p* = 0.001), growth type (*p* < 0.001), mitosis (*p* = 0.016) and melanoma subtype (*p* < 0.001), DN^−^ melanomas were more aggressive compared to melanomas with benign nevus cell islands and DN^+^ melanomas (Supplementary Table 2).

### Immunosuppression in relation to metastasis and other parameters

The patients with immunosuppression showed higher total (30.3% vs. 15.4%, *p* = 0.001) and distal (19% vs. 8.2%, *p* = 0.003) metastasis rate compared to non-immunosuppressed patients, but the difference in the metastasis rate into lymph nodes was not significant (20.6% vs. 13.4%, *p* = 0.088). In the immunosuppressed group, melanoma had metastasized to more than one anatomical location in 12.8% of the subjects, but only in 3.1% of the non-immunosuppressed ones (*p* = 0.001).

Immunosuppressed subjects showed a higher Breslow thickness and Clark level (*p* = 0.018 and *p* = 0.013, respectively) (Table 4) as well as more frequently dysplastic nevi (26.1% vs. 14.4%, *p* = 0.007) compared to non-immunosuppressed subjects.

**Table 4:** The comparison of the main parameters of melanoma between the patients with (IS) and without (non-IS) immunosuppression

	IS (142)	Non-IS (195)	p-value (IS versus non-IS)
**Clark (data from 337 cases)**			
**5**	10.6%	6.7%	0.013
**4**	26.8%	16.4%	
**3**	26.8%	23.1%	
**2**	8.5%	16.4%	
**1**	27.5%	37.4%	
**Breslow (337 cases)**			
**4**	15.5%	10.3%	0.018
**3**	17.6%	11.3%	
**2**	16.9%	11.3%	
**1**	50.0%	67.2%	
**Mitosis count (224 cases)**			
***A lot***	42.9%	32.5%	0.134
***Little***	42.9%	56.3%	
***No Mitosis***	14.3%	11.1%	
**LV/BV/PN (103 cases)**			
***Yes***	2.5%	3.2%	0.843
***No***	97.5%	96.8%	
**Microsatellites (30 cases)**			
***Yes***	55.6%	33.3%	0.255
***No***	44.4%	66.7%	
**Ulceration (337 cases)**			
***Yes***	16.2%	14.4%	0.642
***No***	83.8%	85.6%	
**Growth type (337 cases)**			
**4**	6.3%	6.2%	0.238
**3**	40.8%	31.8%	
**2**	24.6%	24.1%	
**1**	28.2%	37.9%	
**Regression (335 cases)**			
***Yes***	7.1%	5.7%	0.596
***No***	92.9%	94.3%	
**Cell type (101 cases)**			
***Epithelioid***	14.3%	20.3%	0.790
***Spindle cell***	50.0%	39.0%	
***Nevoid***	7.1%	6.8%	
***Mixed***	23.8%	30.5%	
***Other***	4.8%	3.4%	
**Recidivism (337 cases)**			
***Yes***	7.7%	4.6%	0.230
***No***	92.3%	95.4%	

LV/BV/PN = invasion into lymphatic/blood vessels or perineural area; growth type 1 = pure radial growth, 2 = radial growth with concomitant vertical growth, 3 = vertical growth with some concomitant radial growth and 4 = pure vertical growth

### Metastasis rate between genders

Males showed higher metastasis rate compared to females (28.5% vs. 13.9%, *p* = 0.001), and even more so from TANS (40.2% vs. 17.8%, *p* = 0.009) and HN (21.2% vs. 5.9%, *p* = 0.012) regions. Also, trunk lesions showed a higher lymph node metastasis rate in males (28.2% vs. 6.9%, *p* = 0.019). Males also had a higher lymph node metastasis rate in general (22.9% vs. 9.2%, *p* = 0.001).

The difference between males and females was pronounced in the non-immunosuppressed group (24.5% vs. 6.2%, *p* < 0.001, respectively), especially with regard to lymph node (22.8% vs. 4.3%, *p* < 0.001), but not distal (11.2% vs. 5.2%, *p* = 0.123), metastasis. No significant difference was seen in the immunosuppressed group between genders.

## Discussion

According to the Finnish Cancer Registry, the age-standardized mortality rate (2013–2017) was relatively high especially in the hospital district of Northern Savo (Table 1). It is noteworthy for the interpretation that, in 2010, the treatment of metastatic melanoma was improved by BRAF inhibitors, and in the following years by immunotherapy. However, when comparing the morbidity and mortality between different university hospital regions in 2013–2017 it has to be assumed that the treatment of metastatic melanoma was sufficiently similar according to national guidelines regardless of the university hospital. Based on the regional referral policy, the major part of patients with melanoma are likely to be treated at Kuopio University Hospital, even after initial biopsying or excision of melanoma elsewhere. Therefore, the melanoma material (*n* = 543) collected at Kuopio University Hospital during 2000–2013 and subsequently transferred to the Biobank of Eastern Finland represents reasonably well the melanoma subjects in this hospital district. According to the Finnish Cancer Registry, there were approximately 575 melanomas reported from the hospital district of Northern Savo during January 1st, 2000, through August 31st, 2013.

The present study shows that especially the malignant type of melanoma increased in the Biobank material during 2000–2013 whereas the in situ types did not reveal similar increase. Furthermore, an unexpectedly high proportion of NM and LM/LMM, but a relatively low proportion of SSM, was noted in 337 subjects. Based on the literature, SSM is the most common subtype in Europeans (70%), whereas the proportion of NM and LM/LMM is approximately 10% [10]. In a Norwegian study, SSM was the most frequent subtype (68.2%), followed by NM (25.6%), LM (3.8%) and ALM (1.8%) [11], and in a Danish study performed in the 80’s, the percentages of SSM (72%) and NM (18%) were similar [12]. A Swiss study showed slightly lower figures for SSM (41%), followed by NM (16%) and LMM (14%) [13]. Nevertheless, the distribution of melanoma subtypes is largely dependent on the geographical area with different populations [10]. The distinction between NM and SSM can be challenging in borderline cases, and it is made by determining lateral extension within the epidermis defined as the epidermal component extending more than three rete ridges lateral to the dermal component [14, 15], which may cause bias to the classification.

In comparison to previous Scandinavian studies [11, 16, 17], the melanomas in the present study seemed to be more aggressive in terms of Breslow thickness and Clark level. The Breslow thickness in previous studies showed that 52–56%, 21–22%, 14–15% and 9–11% of the melanomas belonged to Breslow classes 1–4, respectively (in situ melanoma excluded) [11, 16, 17], whereas the corresponding percentages in the present study were 40, 20, 21 and 19%. The percentage of samples with a Clark level 2 through 5, mentioned in two studies [11, 16], was 29–31%, 36%, 29–30% and 4–5%, respectively, whereas in this study the corresponding percentages were 20, 37, 31 and 12%. Also, 22% of the samples were ulcerated in this study, but only 15–19% in the previous studies [11, 16]. It is noteworthy, however, that there may be some selection bias towards more aggressive melanomas in this study, since the most representative samples were included.

The ratio of male to female subjects was 1.03–1.13 that parallels previous findings on the incidence ratio of 1.02–1.1 [3] (https://www.wcrf.org/dietandcancer/cancer-trends/skin-cancer-statistics). Worldwide, the median age at the time of melanoma diagnosis is 57 years [18], and the mean age in the non-Hispanic white population is 54.7–62.1 years (SEER, USA) [19]. In large Swiss and Swedish studies, the median age at diagnosis was 60.3 and 61 years, respectively [13, 16]. In a Norwegian study conducted due to the highest mortality rate of melanoma in Europe, the mean age at diagnosis was 65 years for males and 62 for females [11]. Therefore, the melanoma patients in this study were relatively old, that is, a mean of 66.2 years in 382 patients with MM. Although an inverse association between the age and metastasis was observed, the mortality from melanoma is known to increase with age, and possible age-related mechanisms have been discussed previously [20, 21]. One explanation for the lower observed metastasis rate in the elderly might be that these patients do not undergo sentinel lymph node biopsying as often as younger patients. Alternatively, the patients may have other comorbidities, as suggested by the high morbidity index in the hospital district (Table 1).

Concordant with previous studies, the trunk was the most common body site in males. Interestingly, HN followed by LL were common sites in females, in a contrast to LL in previous studies [12, 22, 23]. One possibility for frequent melanomas in the HN region in females is the relatively high mean age. These two common body sites of melanoma, trunk in males and LL in females, seems to be constant in countries with large latitude variation [24] and in Scandinavia [12, 22, 23]. Nevertheless, the genderwise body site distribution parallels that reported in a previous Finnish study (1953–2003) [24].

Males showed over 2-fold higher metastasis rate compared to females. This may be related, in part, to the result that males had more trunk melanomas, while females more HN melanomas, concordant with previous studies [25, 26]. It seems that melanomas arising on sun-exposed skin exhibit lower angiogenic and lymphangiogenic potential and metastasis rate, and better prognosis than those on the skin without signs of chronic sun-induced damage [27]. However, Fadaki et al. reported that HN melanomas show a significantly increased risk of recurrence and death compared to other body sites [26]. In addition, behavioral differences [24, 28] might play a role between genders. Also, melanomas in TANS have a higher metastasis rate [29] paralleling this study. The prevalence of TANS melanomas was significantly higher, and they also seemed to be more aggressive and prone to metastasize, in males than females (Supplementary Table 1). Thus, TANS melanomas may be considered even more aggressive in males than previously thought.

The immunosuppressive state has been known to worsen the prognosis of melanoma [30–33], and we found that an assumed immunosuppressive state associates with more aggressive melanoma in terms of Breslow thickness, Clark level and metastasis rate. Even though a true immunosuppressive state in these patients could not be confirmed with certainty, even this classification of patients to immunosuppressed and non-immunosuppressed groups revealed a relation between immunosuppression and melanoma.

In summary, the main results of this study are that: 1) especially the samples from the MM type showed the highest increase during 2000–2013; 2) the mean age of patients at diagnosis was relatively high; 3) the proportion of aggressive NMs was relatively high; 4) melanoma metastasis correlated with male gender, younger age, immunosuppressive state, Clark level, Breslow thickness, ulceration, mitosis count, invasion into lymphatic/blood vessels or perineural area, the presence of microsatellites, melanoma subtype, body site, recidivism and DN^−^; 5) males showed over 2-times higher metastasis risk compared to females, and this may be related to the high prevalence of trunk and TANS melanomas in males; and 6) immunosuppression associated with more aggressive melanoma. These findings can explain, in part, the relatively high mortality rate from melanoma during 2013–2017 and prompts enhanced targeted screening of melanoma in the population. Furthermore, the study results can be generalized to demonstrate how a tissue material in biobank can aid in finding explanations to health issues in population.

## Supplementary Information

**Additional file 1: Supplementary Table 1.** Comparison between melanomas of thorax, upper arm, neck and scalp (TANS) and those outside TANS region (non-TANS) in respect to nodular melanoma (NM) prevalence and metastasis rate. **Supplementary Table 2.** Comparison of the main parameters between melanomas with no dysplastic nevus cells (DN^−^), melanomas with benign nevus cell islands and melanomas with dysplastic nevus cells (DN^+^, possible origin from nevus), only significant results shown.

## Data Availability

The datasets generated and/or analysed during the current study are available from the corresponding author on reasonable request. All major data generated or analysed during this study are included in this published article [and its supplementary information files].

## Abbreviations

ALM: acral lentiginous melanoma
LM/LMM: lentigo maligna/lentigo maligna melanoma
NM: nodular melanoma
SSM: superficial spreading melanoma
HN: head & neck
UL: upper limb
LL: lower limb
GPA: genitalia/perianal area
TANS: thorax, upper arm, neck and scalp
DN^+^: melanoma with dysplastic nevus cells
DN^−^: melanoma without dysplastic nevus cells
MIS: melanoma in situ
MM: malignant melanoma
