# Supplementary material for: High regional mortality due to malignant melanoma in Eastern Finland may be explained by the increase in aggressive melanoma types
Source: BMC Cancer. 2021 Oct 29;21:1155. doi: 10.1186/s12885-021-08879-1 (PMC8555296; doi:10.1186/s12885-021-08879-1)
Supplement: Supplementary file 1 — Additional file 1: Supplementary Table 1. Comparison between melanomas of thorax, upper arm, neck and scalp (TANS) and those outside TANS region (non-TANS) in respect to nodular melanoma (NM) prevalence and metastasis rate. Supplementary Table 2. Comparison of the main parameters between melanomas with no dysplastic nevus cells (DN−), melanomas with benign nevus cell islands and melanomas with dysplastic nevus cells (DN+, possible origin from nevus), only significant results shown. [file 12885_2021_8879_MOESM1_ESM.docx]

Supplementary table 1. Comparison between melanomas of thorax, upper arm, neck and scalp (TANS) and those outside TANS region (non-TANS) in respect to nodular melanoma (NM) prevalence and metastasis rate.

|  | **non-TANS**  **(136)** | **TANS**  **(116)** | **p-value** |
| --- | --- | --- | --- |
| **NM prevalence** | 30.1% | 43.1% | 0.033 |
| **Lymph node metastasis** | 17.4% | 28.6% | 0.041 |
| **Distal metastasis** | 12.5% | 22.4% | 0.037 |
| **Lymph node or distal metastasis** | 20.6% | 38.8% | 0.001 |
| **Prevalence, male** | 40.9% | 59.1% | <0.001 |
| **Prevalence, female** | 68.3% | 31.7% |  |
| **Lymph node or distal metastasis (male)** | 25.9% | 47.4% | 0.013 |
| **Lymph node or distal metastasis (female)** | 17.1% | 21.1% | 0.6 |

Lentigo maligna and lentigo maligna melanoma were excluded from the analysis, since this type of melanoma would be mainly in the non-TANS region and would affect the results so that the difference in the proportion of NM and metastasis rate between TANS and non-TANS would increase. The definite anatomical site was available for 325 melanomas for determining the melanomas in TANS and non-TANS region.

Supplementary table 2. Comparison of the main parameters between melanomas with no dysplastic nevus cells (DN^-^), melanomas with benign nevus cell islands and melanomas with dysplastic nevus cells (DN^+^, possible origin from nevus), only significant results shown.

|  | | **DN^-^ (44)** | ***Benign nevus cell islands* (34)** | **DN^+^ (68)** | **p-value** |
| --- | --- | --- | --- | --- | --- |
| **Metastasis** | ***Yes*** | 33.8% | 26.5% | 4.5% | 0.001 |
|  | ***No*** | 66.2% | 73.5% | 95.5% |  |
| **Breslow** | **4** | 33.8% | 2.9% | 0.0% | <0.001 |
|  | **3** | 7.4% | 8.8% | 6.8% |  |
|  | **2** | 17.6% | 20.6% | 2.3% |  |
|  | **1** | 41.2% | 67.6% | 90.9% |  |
| **Clark** | **5** | 17.6% | 0.0% | 2.3% | <0.001 |
|  | **4** | 30.9% | 29.4% | 0.0% |  |
|  | **3** | 30.9% | 35.3% | 18.2% |  |
|  | **2** | 20.6% | 14.7% | 11.4% |  |
|  | **1** | 0.0% | 20.6% | 68.2% |  |
| **Ulceration** | ***Yes*** | 30.9% | 8.8% | 4.5% | 0.001 |
|  | ***No*** | 69.1% | 91.2% | 95.5% |  |
| **Growth type** | **4** | 5.9% | 2.9% | 2.3% | <0.001 |
|  | **3** | 61.8% | 35.3% | 9.1% |  |
|  | **2** | 32.4% | 41.2% | 20.5% |  |
|  | **1** | 0.0% | 20.6% | 68.2% |  |
| **Mitosis count** | ***A lot*** | 54.5% | 38.5% | 17.4% | 0.016 |
|  | ***Little*** | 31.8% | 53.8% | 56.5% |  |
|  | ***No*** | 13.6% | 7.7% | 26.1% |  |
| **Body site** | **Head & neck** | 19.4% | 20.6% | 47.7% | 0.044 |
|  | **Trunk** | 40.3% | 47.1% | 34.1% |  |
|  | **Upper limb** | 19.4% | 11.8% | 11.4% |  |
|  | **Lower limb** | 10.4% | 14.7% | 6.8% |  |
|  | **Foot & sole** | 6.0% | 2.9% | 0.0% |  |
|  | **Palm** | 4.5% | 0.0% | 0.0% |  |
|  | **Genitalia/perianal** | 0.0% | 2.9% | 0.0% |  |
| **CMM subtype** | **NM** | 57.4% | 38.2% | 11.4% | <0.001 |
|  | **ALM** | 10.3% | 2.9% | 0.0% |  |
|  | **SSM** | 32.4% | 50.0% | 29.5% |  |
|  | **LM/LMM** | 0.0% | 8.8% | 59.1% |  |

Growth type 1 = pure radial growth, 2 = radial growth with concomitant vertical growth, 3 = vertical growth with some concomitant radial growth and 4 = pure vertical growth; NM = nodular; ALM = acral lentiginous; SSM = superficial spreading; and LM/LMM = lentigo maligna/lentigo maligna melanoma.
